# Spaced Scenario Demonstrations Improve Knowledge and Confidence in Pediatric Acute Illness Management

**DOI:** 10.3389/fped.2014.00133

**Published:** 2014-11-24

**Authors:** Rahul Ojha, Anthony Liu, Bernard Linton Champion, Emily Hibbert, Ralph Kay Heinrich Nanan

**Affiliations:** ^1^Sydney Medical School-Nepean, The University of Sydney, Sydney, NSW, Australia; ^2^Schulich School of Medicine and Dentistry, University of Western Ontario, London, ON, Canada

**Keywords:** medical education, simulation, knowledge retention, spaced education

## Abstract

**Objectives:** Nationally accredited simulation courses such as advance pediatric life support and pediatric advance life support are recommended for health care professionals (HCPs) at two yearly intervals as a minimum requirement, despite literature evidence suggesting rapid decline in knowledge shortly after course completion. The objective of this study was to evaluate an observation-based, educational intervention program aimed at improving previously acquired knowledge and confidence in managing critical illnesses.

**Methods:** A prospective cohort longitudinal study was conducted over a 6-month period. Participants were assessed with a knowledge based questionnaire immediately prior to and after observing 12 fortnightly critical illness scenario demonstrations (CISDs). The outcome measure was performance on questionnaires. Regression analysis was used to adjust for potential confounders. Questionnaire practice effect was evaluated on 30 independent HCPs not exposed to the CISDs.

**Results:** Fifty-four HCPs (40 doctors and 14 nurses) participated in the study. All participants had previously attended nationally accredited simulation courses with a mean time since last attendance of 1.8 ± 0.4 years. The median number of attendances at CISD was 6 (2–12). The mean questionnaire scores at baseline (17.2/25) were significantly lower than the mean post intervention questionnaire scores (20.3/25), *p* = 0.003. The HCPs self-rated confidence in managing CISD was 6.5 times higher at the end of the program in the intervention group (*p* = 0.002) than at baseline. There was no practice effect for questionnaires demonstrated in the independent sample.

**Conclusion:** The educational intervention program significantly improved the knowledge and confidence of the participants in managing pediatric critical illnesses. The CISD program provides an inexpensive, practical, and time effective method of facilitating knowledge acquisition and retention. Despite the distinctively different approach, this study has shown the effectiveness of the participant being an observer to enhance pediatric resuscitation skills.

## Introduction

Nationally accredited resuscitation simulation courses such as advance pediatric life support (APLS) and pediatric advance life support (PALS) have been broadly implemented into clinical training schemes and are important elements of continuing medical education programs (1–4). There is evidence for an immediate positive effect of these accredited resuscitation simulation courses on medical knowledge, skills, and confidence (5–7).

There is also little data on improved patient outcomes based on participation in resuscitation simulation courses. Zendejas et al. found in a meta analysis that simulation-based education was associated with small to moderate patient benefits in comparison with no intervention and non-simulation instruction (8). However, there is a lack of evidence in the literature regarding the improvement in knowledge and confidence of observers of simulation. It is also unknown how passive observation compares to active participation in terms of retention of knowledge and skills.

The recommended interval between undertaking nationally accredited resuscitation simulation courses is 2 years (7, 9–11) with recent recommendations for even more frequent refresher courses at six monthly intervals (12).

It can be speculated that these intervals are based on practical reasons. These include (a) the requirement for regular updates due to the emergence of new evidence impacting on clinical practice; (b) the extensive demand on resources and time involved in running simulation courses; and (c) the intention to maintain an adequate cohort of health care professionals (HCPs) with exposure to these courses.

The interval between courses is critical for maintaining the knowledge and skills essential for improving patient outcomes. Depending on the initial volume, content, and intensity of simulation courses, knowledge, skills, and confidence levels have been shown to decline within a few weeks to several months after course completion (12–22). This calls for novel strategies to retain and reactivate knowledge acquired in medical simulation courses through practical, inexpensive, and time efficient teaching programs.

Since it is essential that HCPs learn and retain the skills for managing pediatric emergency situations, we designed a spaced education critical care scenario management program to meet this need. “Spaced education” refers to educational programs that are designed to enhance retention of knowledge utilizing the so-called spacing effect. The spacing effect refers to the finding that education encounters, which are spaced and repeated over time (spaced distribution) result in more efficient and improved retention of learning, compared with more concentrated educational encounters, referred to as bolus education (23–25).

In this study, we present the impact of observing routinely scheduled scenario demonstrations of critical pediatric illness scenarios in a public hospital setting by pediatric medical and nursing staff.

## Materials and Methods

This was a prospective, interventional, longitudinal study conducted between June and November 2012 in a general metropolitan tertiary teaching hospital, with a busy emergency department seeing more than 40,000 presentations per year and housing its own intensive care unit. Study participants were pediatric HCPs who attended weekly Pediatrics Grand Rounds as part of the departmental timetable. The attendance of the participants was recorded and tracked. Fifteen minutes at the beginning of Grand Rounds were allocated on a fortnightly basis to scenario demonstrations. The same scenarios were also held at the same frequency for the pediatric nurses on the ward at other times during their routine patient handover session. Each participant provided written consent and their questionnaire results were de-identified using a unique identification code.

Ethics approval was obtained through the Nepean Blue Mountains Local Health District Human Ethics Committee (Study no. LNR/12/Nepean/68).

Six scenarios were designed using a modified Delphi method. Authors developed a consensus among a panel of experts utilizing the available national and international resuscitation guidelines 26–29 (Table 1). A panel of six experts participated in a modified Delphi panel for achieving convergence of opinion. A two-thirds majority was used for consensus building from responses obtained by these experts to finally develop the six scenarios. All six scenarios were piloted before the intervention. A group (2–4) of pediatric year 4 resident doctors participated in the trial sessions each week before the actual intervention. Each scenario was piloted once and was modified based on feedback from participating residents. These residents were excluded from the study.

**Table 1:** **Simulated critical case descriptions**.

	Case type	Clinical presentation
1	Acute severe asthma	Severe respiratory distress resulting in respiratory arrest
2	Status epilepticus	Prolonged generalized tonic–clonic seizure, non-responsive to benzodiazepines and barbiturates
3	Apneic newborn	Term baby born pale, hypotonic, and unresponsive
4	Anaphylaxis	Generalized urticarial rash with breathing difficulties, resulting in cardio-respiratory arrest
5	Septic shock	Febrile, lethargic, and mottled toddler with a non-blanching rash, resulting in cardio-respiratory arrest
6	Supraventricular tachycardia	Young child with dizziness becoming drowsy with altered sensorium

Checklists were developed for each scenario from the PALS and APLS curriculum. The scenarios and checklists were developed by a panel of pediatricians with input from pediatric critical care experts. Each checklist covered criteria including important items of history, physical exam, diagnosis, and management, as well as skill components of the case such as cardioversion and intubation. The cardiopulmonary resuscitation (CPR) checklist and the intubation checklist were standardized across all scenarios. The CPR checklist covered all five components of high-quality CPR; adequate rates, depth, complete chest recoil, minimal interruption, and avoidance of excessive ventilation. The intubation checklist assessed items such as preparation, preoxygenation, cricoid pressure, sedation, and postintubation assessment of proper endotracheal tube placement. During the scenario demonstration session, the checklist was completed by one of the investigators with the intention to ensure that all the tasks were completed for each scenario in accordance with the resuscitation guidelines. Steps missed in any session were discussed in the debriefing session.

The scenarios included initial clinical vignettes, which were read out to the participants. Based on the vignette, investigators then managed the case according to the information provided and in alignment with the current resuscitation guidelines. The study participants observed investigators perform the entire task. Investigators demonstrated high-quality CPR during the scenario demonstrations including the entire five quality components.

For this purpose, an age appropriate low-fidelity mannequin and all other necessary equipment were used for the practical demonstration. A whiteboard was used to list medication dosing and equipment sizing. To provide maximum exposure in handling critical care patients all scenarios culminated in an end point of cardiac, respiratory, or cardio-respiratory arrest.

Each scenario took approximately 10 min followed by 5 min of debriefing. The debriefing sessions were run in a standardized fashion to provide specific feedback aimed at improving the performance. This was in the form of a dialog with participant involvement. Feedback was tailored to the learning objectives such as knowledge, technical and communication skills, and team behavior. Special focus was on quality of CPR in the feedback sessions.

The six scenario demonstrations were held fortnightly at Pediatric Grand Rounds. Only one scenario was demonstrated at each Grand Round and then each scenario was repeated once over the next 3 months so that the course of scenarios was completed over 6 months. All Pediatric Grand Rounds attendees who participated in the study were HCPs (residents, pediatricians, and nurses). Participants attended according to their roster. If rostered for the day, then they attended grand rounds, attendance at which is obligatory.

**Figure 1:**
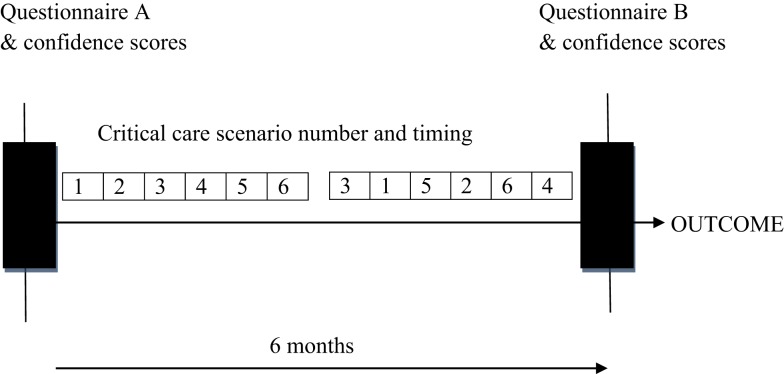
**Six scenarios were held fortnightly and then each was repeated once over the next 3 months**. Participants were assessed by two questionnaires (A and B), one initially and one 6 months after completion of scenario sessions.

The impact of the education intervention on the participants who attended Pediatric Grand Rounds was assessed by having participants complete the two questionnaires (A and B) in a written format at the Pediatric Grand Rounds where the scenarios were demonstrated. The first questionnaire was completed prior to the commencement of scenario demonstrations (Questionnaire A) and the second 6 months later (Questionnaire B), which was 2 weeks after completion of scenario demonstrations (Figure 1). The participants’ attendance at Pediatric Grand Rounds was tracked and recorded.

A question bank of 75 pediatric emergency medicine and critical care written multiple-choice questions (MCQ) pertinent to the case scenarios were prepared with the aim to appropriately assess participant’s patient assessment and intervention skills. The questionnaires were derived from the current resuscitation guidelines (APLS and PALS) and were designed and prepared by two pediatric emergency experts (26–29). Each questionnaire (A and B) had questions that aimed to cover acute care pediatrics and had the same content but presented in a different way. The intention was to uniformly assess participants’ theoretical knowledge.

Twenty-five questions were randomly chosen by computer for the initial questionnaire A undertaken by all participants and 25 for the post demonstration questionnaire B. Twenty percent of questions were repeated between the two tests. Thirty minutes were allocated for participants to complete the questionnaires. Participants were not informed of their results at any time during the study.

Participants’ confidence level in managing each whole scenario was assessed at the same time as questionnaire A and B using a Likert scale where 0–2 = not confident, 3–5 = somewhat confident, 6–8 = confident, and 9–10 = very confident. The different Likert scale categories were aggregated into two distinct categories of confident vs. not confident for analysis. The confident category was 6–10 on the Likert scale and not confident category as 0–5 on the Likert scale.

An independent non–intervention group of 30 final year medical students was involved in the study for the purpose of detecting any potential practice effect as a result of completing the questionnaires rather than as a control group for participants. The 30 medical students were given the 2 questionnaires at the same time points as the participants involved in the scenarios without being exposed to scenario demonstrations over the same study time period. Their level of self reported confidence in managing the scenarios was also recorded.

The outcome measure was the difference in pre and post questionnaire scores and self reported confidence levels. This was assessed with paired *t*-test. Data were analyzed using SPSS version 20 (SPSS, Inc., Chicago). Descriptive statistics were reported as means and SD. Comparisons between all categorical measures were made using a Chi-squared test and the Student’s *t*-test for continuous measures. The associations between pre-scenario confidence score, attendance of scenarios, and post-scenario confidence scores were assessed using logistic regression modeling. Both univariate and multivariate analyses were conducted to control for potential confounders, such as time in the profession, professional role, and time since last undertaking a formal simulation course outside the study.

## Results

Eighteen post-graduate trainee year 1–2 (PGY 1–2) doctors, 18 post-graduate trainee year 3–4 (PGY3–4) doctors, 4 pediatricians, and 14 registered pediatrics nurses participated in the study. All HCPs had previously completed nationally accredited simulation courses – PALS, neonatal life support (NLS), and Resus4Kids within the last 8–24 months (1.8 ± 0.4 years). Table 2 describes the demographic details of the HCPs, including their level of experience and time since they last completed a simulation course.

**Table 2:** **Baseline characteristics of participants in the simulation study**.

Profession	Position	Numbers	Sessions attended (out of 12)	Time in profession (years)	Time since simulation (years)
Doctor (*n* = 40)	PGY 1–2	18 (33.3%)	5.7 ± 1.56	1.2 ± 0.42	0.7 ± 0.42
	PGY 3–4	18 (33.3%)	7.4 ± 1.50	3.4 ± 0.51	2.6 ± 1.8
	Pediatrician	4 (7.4%)	4.5 ± 2.60	6.5 ± 0.57^a^	2.2 ± 0.42
Nurse (*n* = 14)		14 (25.9%)	6.1 ± 1.18	4.4 ± 0.17	2.1 ± 1.0

*PGY : post-graduate year following completion of medical school*.

*^a^Time as a pediatrician, not including the prior years of training*.

There was a statistically significant difference in pre and post intervention knowledge questionnaire scores for participating HCPs. Pre-intervention mean score was 17.2 (out of 25) (=69%) and the mean score after the scenario demonstrations was 20.3 (out of 25) (=81%) with *p* = 0.003. Analysis of the results from the non-intervention independent cohort showed no evidence of a practice effect with no improvement in questionnaire scores over time. The baseline mean score in this group was 17.3 (out of 25) and the mean score at the end of the study was 17.1 (out of 25) with *p* = 0.0001.

Participating HCPs also showed improved self-rated confidence levels in managing critical care scenarios as a whole at 6 months following attendance at the scenarios compared with baseline. Thirty-five percent of HCPs were confident at the start of the study increasing significantly to 72% at the end of the study, *p* < 0.001 (Table 3). The analysis of the results from the non-independent cohort showed no evidence of a practice effect with no improvement in the self-rated confidence.

**Table 3:** **Pre- and post-scenario scores and confidence levels**.

	Pre-scenario	Post-scenario	*p*-Value
Questionnaires mean score out of 25 (%)	17.2 (68.8%)	20.3 (81.2%)	0.003
Confidence levels^a^ of 44 HCP’s (%)
Confident	19 (35.2%)	39 (72.2%)	<0.001
Not confident	35 (64.8%)	15 (27.8%)	

*The different Likert scale categories are aggregated into two distinct categories of confident vs. not confident*.

*^a^Confident = 6–10 on Likert scale, not confident = 0–5 on Likert scale*.

Univariate analysis was performed on potential confounders such as time in the profession, professional role, and time since last simulation course was completed. Pre-confidence score was the only parameter, which had a positive significant effect on post confidence scores. The odds of an HCP rating his or herself as confident in managing the critical illness scenario demonstrations (CISDs) was 6.5 times higher at the end of the program in the intervention group than for HCPs who were not exposed to the CSID (*p* = 0.002). There was significant benefit from repeated sessions (*p* = 0.014) (Table 4).

**Table 4:** **Regression analysis of post-scenario confidence levels**.

Parameter	OR	95% CI	*p*-Value
Attendance (yes/no)	6.54	1.90–22.55	0.002
Number of attendances	3.35	1.27–8.82	0.014

*Adjusted for pre-scenario confidence scores*.

## Discussion

This prospective study found that the implementation of a program of repeated observation of brief CISD spaced over time provided an effective method for improving knowledge and increasing confidence among pediatric HCPs managing critically ill children. Improvements in confidence of the HCPs in managing the brief critical illness scenarios occurred in tandem with improved knowledge. This is the first study describing the educational impact of participant observation of demonstrations of simulated pediatric critical illness scenarios as opposed to the more usual active participation in running simulations of pediatric critical illness scenarios.

The CISD program utilized in this study is inexpensive and can easily be implemented as a teaching program in the hospital setting. It requires only a low-fidelity mannequin and a small amount of resuscitation equipment and does not need to be run in simulation centers. Once developed, it can be used repeatedly as it covers the main pediatric critical illness scenarios encountered in practice. It requires two to four clinically competent staff to run the demonstrations, debriefing, and feedback and can be incorporated into routinely scheduled department meetings, which the relevant target audience are already likely to attend. The program has the advantage of being relevant and appropriate for a wide range of HCPs.

Although critical illness scenarios have been tested as an isolated intervention in experimental settings, incorporation into a routine teaching curriculum over a longer-time span has not been trialed to our knowledge. With this design, it was therefore possible to look at the intra-individual effect on improvement in and retention of knowledge. In contrast to previous studies, the design of this study has the advantage of being able to assess retention of knowledge of acute illness scenarios over a longer period of time where opportunities for repeated reinforcement of knowledge are provided as opposed to a single brief education intervention.

Several studies have involved development of a brief education course with delivery of few critical care scenarios on a single day and testing of knowledge and skills at the conclusion of the course. These studies have shown significant improvement in knowledge, skills, and confidence in management of these scenarios. However, the testing was done immediately after the course (20, 30–33). Few studies have looked at the long-term impact of these brief education interventions on knowledge and skills (17, 33–37). Skidmore et al. investigated long-term effects on knowledge and skill retention after a neonatal resuscitation course (17). In this study, a cohort of 62 participated in the resuscitation course and was tested before and soon after the course. Their theoretical and practical skills were retested 6 and 12 months after course completion. There was significant improvement immediately after the course that was maintained over the following 6 months and then declined. However, it has to be acknowledged that there would have been practice effect on neonatal resuscitation in a neonatal setting, which would not apply to the CISD. In the pediatric setting, the management of these scenarios is performed infrequently.

Such a decline in knowledge is not unexpected, as forgetting is a natural psychological phenomenon. This can be overcome by spacing of the education over time. Psychological research studies have also shown that, by repeated reinforcement of learning there is improvement in retention over time (38–40).

Many studies using the spaced education method have demonstrated improved retention of knowledge (41–44). One study demonstrated that spaced education consisting of repetitive case scenarios and clinical questions spaced every week over a 1 year time period significantly improved third year surgical students’ retention of medical knowledge (45).

The results of our study are encouraging. Employing the spacing effect could be applied to medical education in general. However, there are some questions that need further attention, such as the optimal spacing patterns to facilitate retention and learning and the number of repetitions required.

Strengths of the study were that it was performed in a real world setting and that participants were mainly those HCPs most likely to be initial responders in the event of pediatric critical illness situations.

There are some limitations to this study. The sample size was limited due to the availability of those attending Grand Rounds. Hence, a larger multicentre, study design would be required to consolidate this approach and determine the longer-term effects of this intervention. Pre and post education intervention knowledge was assessed rather than assessing performance of practical skills as a member of a tea. It is uncertain whether improved knowledge would be associated with improved performance in the workplace.

The other potential limitation was that the comparison cohort comprised final year medical student with more limited experience than the HCPs. However, surprisingly, the HCPs did not perform better at baseline than these final years medical students, suggesting that it was an adequate comparison group. All simulation courses cover similar competencies. Despite the fact that all HCPs in the intervention group had undertaken simulation courses in the past they performed no better on a knowledge test at baseline than final year medical students who are midway through their pediatric rotation.

It remains unknown how long the improvement in knowledge will persist following an intervention such as ours. Our intention is to continue practice at regular intervals as Skidmore et al. found that even in a setting where professionals underwent a long course, the theoretical, and practical skills were maintained for 6 months after the course but then started to decline (17).

## Conclusion

Despite the distinctively different approach, this study has shown the effectiveness of the participant being an observer to enrich pediatric resuscitation skills.

Our study has shown that a simple educational intervention program based on repeated observation of brief critical scenario demonstrations has significantly improved the knowledge and confidence of HCPs in managing pediatric critical illnesses. However, it remains unknown how long the improvement in knowledge will persist following such an intervention and hence a larger multicentre, study design would be required to consolidate this approach and determine the longer-term effects of this intervention.

Such an educational innovation can be easily incorporated into a training curriculum. This educational program has the benefits of being time efficient and inexpensive. It requires minimal resources to run and thus can be made available to a wide range of HCPs. Further research is required to assess whether this program improves HCPs performance in managing pediatric critical illness scenarios in the workplace and improves patient outcomes.

## Conflict of Interest Statement

The authors declare that the research was conducted in the absence of any commercial or financial relationships that could be construed as a potential conflict of interest.
